# Comparative Metabolomics of *Clostridium acetobutylicum* ATCC824 and its Engineered Strain, *C. acetobutylicum* DG1

**DOI:** 10.4014/jmb.2407.07028

**Published:** 2025-02-25

**Authors:** Jae Hyuk Chung, Jieun Lee, Sooah Kim, Kyoung Heon Kim

**Affiliations:** 1 Department of Biotechnology, Graduate School, Korea University, Seoul 02841, Republic of Korea; 2 Department of Environmental Science and Biotechnology, Jeonju University, Jeonju 55069, Republic of Korea

**Keywords:** *Clostridium acetobutylicum* ATCC824, *C. acetobutylicum* DG1, genetic engineering, metabolomics

## Abstract

*Clostridium acetobutylicum*, a strict gram-positive anaerobe, plays a pivotal role in biotechnological applications, particularly in the biosynthesis of 1,3-propanediol, a critical biofuel component and monomer for bioplastic production. This study introduces *C. acetobutylicum* DG1, a metabolically engineered strain designed to enhance the 1,3-propanediol pathway. Despite its development, comprehensive metabolic comparisons between the parent and modified strains remain unexplored. Our research addresses this gap by employing gas chromatography coupled with time-of-flight mass spectrometry to delineate the global metabolite landscapes of both strains. Through multivariate statistical analysis such as principal component analysis and hierarchical clustering analysis, we discovered pronounced disparities in their metabolite profiles across the acidogenic and solventogenic phases. Detailed metabolomics investigations underscored significant divergences in amino acid metabolism, fatty acid metabolism, and the tricarboxylic acid cycle. These findings shed light on the metabolic alterations induced by genetic engineering in *C. acetobutylicum*, offering novel insights into microbial metabolism that could guide future biotechnological innovations.

## Introduction

Most of energy utilized is derived from fossil fuels, and the increased use of these fuels accelerates global warming, leading to further environmental challenges [1-3]. Various studies have been performed to solve the problem of global warming, and it is particularly important to secure alternative energy sources that do not use fossil fuels [4-6]. Alternative fuels obtained via microbial fermentation are easily accessible and usable.

In 1861, Louis Pasteur discovered butanol production in *Clostridium* cultures; consequently, *Clostridium* became an excellent strain for solvent production [7]. In the early 1900s, Weismann used a wild type strain of *Clostridium acetobutylicum* for acetone and butanol production at the industrial scale [8-11]. *C. acetobutylicum*, a promising and industrially useful strain, is a gram-positive, strictly anaerobic bacterium that uses multiple substrates for acetone–butanol–ethanol (ABE) fermentation to produce acetone, butanol, and ethanol [12]. ABE fermentation by *C. acetobutylicum* ATCC824 is divided into the following two phases: acidogenesis and solventogenesis [13, 14]. Acidogenesis is the rapid accumulation of organic acids (butyric and acetic acids) and the production of hydrogen and carbon dioxide during exponential growth, which results in a pH drop to 4.5 [15]. Solventogenesis is the reassimilation of organic acids from acidogenesis to produce ethanol and butanol, which slows the growth rate of the strain [9, 16-18]. Conversely, the engineered strain *C. acetobutylicum* DG1 lacks genes required for acetone and butanol formation, and it produces 1,3-propanediol as its main fermentation product.

1,3-Propanediol has been used in the industrial production of various goods such as fuels, biopolymers, cosmetics, and foods [19-21]. Several microbial species, such as *Lactobacillus buchnerii*, *Bacillus welchii*, *Klebsiella pneumonia*, and *Clostridium butyricum*, have been used for the biological production of 1,3-propanediol from glycerol [22, 23]. These bacterial species possess metabolic pathways necessary for glycerol-to-1,3-propanediol conversion. *C. acetobutylicum* cannot use glycerol as a carbon source because it cannot reoxidize the excess nicotinamide adenine dinucleotide hydrogen produced by glycerol catabolism. Therefore, an engineered strain, *C. acetobutylicum* DG1, into which the pSPD5 plasmid possessing the 1,3-propanediol pathway was introduced, has been developed.

Metabolomics is the study of global changes in the entire set of metabolites in an organism. Metabolomics data provide valuable information, such as the physiological and metabolic status of a cell based on changes in metabolite levels caused by genetic or environmental perturbations. Thus, metabolomics is a powerful tool to determine the metabolism and physiology of *C. acetobutylicum*. However, only a few studies have used this approach to investigate the action mechanisms of *C. acetobutylicum* DG1. Thus, herein, the global metabolite profiles of *C. acetobutylicum* ATCC824 and its engineered strain *C. acetobutylicum* DG1 were established to identify the metabolic characteristics, biological functions, and mechanisms of the engineered strain DG1.

## Materials and Methods

### Bacterial Strains and Growth Conditions

*Clostridium acetobutylicum* ATCC824 and its engineered mutant strain DG1 (pSPD5), obtained from Institut National des Sciences Appliquées (INSA; France), were anaerobically cultivated in a reinforced clostridial medium (Difco Laboratories, USA) at 37°C [24]. Cell growth and pH were monitored by measuring the optical density at 600 nm and calibrating against the dry weight of the cell.

### Metabolite Extraction and Analysis

Metabolite extraction and analysis were performed by slightly modifying a previously described method [25, 26]. A cell culture (1 ml) was harvested by vacuum filtration using a nylon membrane filter (0.20 μm pore size, 30 mm diameter; Whatmann, USA) and washed with distilled water. The filter contacting the cells was immediately submerged into 10 ml of an extraction solvent (acetonitrile:methanol:water = 2:2:1) at −20°C, followed by immersion in liquid nitrogen. The procedure was completed within 30 s and performed inside an anaerobic chamber. After thawing, the extraction mixture was vortexed for 3 min and centrifuged at 16,100 g for 5min at 4°C. The supernatant was collected and completely dried using a vacuum concentrator (Labcono, USA). The extract was re-extracted with 50ACN (acetonitrle:water = 1:1) to remove lipids and waxes at 0°C. The re-extraction was subjected to the same steps mentioned in the previous sentence. The extract metabolites were derivatized with 5 μl of 40 mg ml^−1^ methoxyamine hydrochloride in pyridine (Pierce, USA) for 90 min at 30°C and 45 μl of *N*-methyl-*N*-trimethylsilyltrifluoroacetamide (Fluka, Switzerland) for an additional 30 min at 37°C. A mixture of fatty acid methyl esters (C8-C30) was added to each extract as an internal retention index marker. Metabolite profiling with gas chromatography/time-of-flight mass spectrometry (GC/TOF MS) was performed. The 1-μl aliquot of the derivatized extract was injected splitless into an Agilent 7890 GC (Hewlett-Packard, USA) equipped with the RTX-5Sil MS capillary column with a length of 30 m, a film thickness of 0.25 mm, and an inner diameter of 25 mm (Restek, USA) and additional 10-m long integrated guard column. The column temperature was maintained at 50°C for 1 min, after which it was increased by 20°C min^−1^ to 330°C at 20°C and maintained for 5 min. The column effluent was injected into the ion source of the Pegasus HT-TOF MS system (USA). The transfer-line temperature was set at 280°C. Ions were generated at a potential of 70 eV, a temperature of 250°C using an ion source, and 10 spectra s^-1^ were acquired in the mass range 85–500 mz^−1^.The GC/TOF MS was autotuned using three ions (mz^-1^ 69, 219, and 502) from perfluorotributylamine spectrum. A mixture of 29 pure compounds including alanine, glutamate, glucose, and cholesterol were analyzed per ten samples for quality control. Six independent replicate samples were extracted and analyzed.

### Statistical Analysis

The Leco Chroma TOF software (version 3.34; USA) was used to preprocess the GC/TOF MS data using automated peak detection and mass spectral deconvolution. To identify the metabolites of *C. acetobutylicum*, the preprocessed data were further processed using Binbase, an in-house programmed database, as previously described [27, 28]. The raw data were normalized to the dry weight of the cell for each sample. For univariate and multivariate statistical analyses, the normalized data were introduced into the Statistica software (version 7.1, StatSoft, USA) [29, 30]. MultiExperiment Viewer was used for hierarchical clustering analysis (HCA) to visualize and organize the metabolite profiles of the two strains [31]. To evaluate changes in the metabolites and metabolism of the two strains, a Student’s *t*-test was used, and pathway analysis with a false discovery rate-adjusted p-value threshold of 0.05 was performed using MetaboAnalyst 5.0 (http://www.metaboanalyst.ca).

## Results

### Growth of *C. acetobutylicum* ATCC824 and the Engineered Mutant Strain DG1

**Fig. 1:**
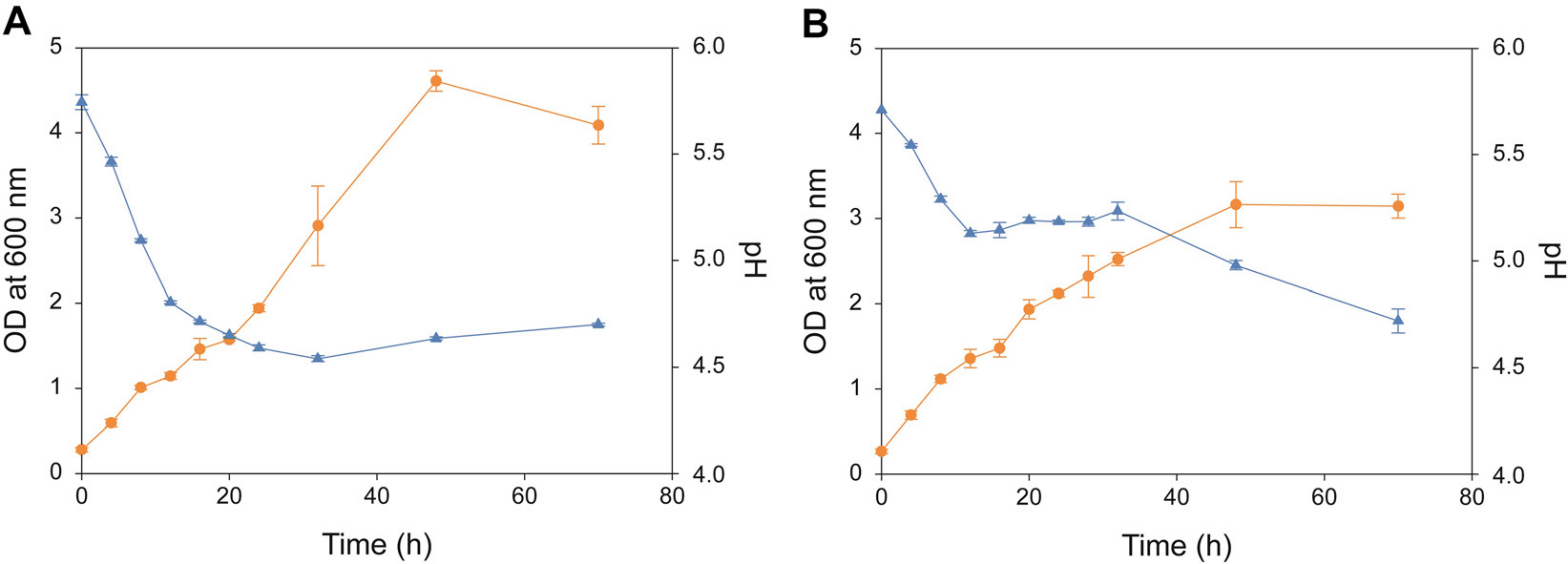
Cell growth (organe color) and pH (blue color) of (A) *Clostridium acetobutylicum* ATCC824 (wild type) and (B) *C. acetobutylicum* DG1 (engineered strain).

The growth profiles of *C. acetobutylicum* ATCC824 (wild type) and DG1 (DG1 mutant) were similar at certain time points; however, the wild type showed a higher growth rate than the mutant in the later stages. Additionally, their exogenous pH profiles differed significantly according to cultivation time (Fig. 1). The cell growth of the wild type strain increased during solventogenesis; however, the growth rate was slower than that during acidogenesis. The exogenous pH profile of the wild type strain decreased and increased during the acidogenic and solventogenic phases, respectively. However, no separate acidogenic and solventogenic phases were observed in fermentation using the DG1 mutant because of the continuous decrease in the exogenous pH profile of the strain. The observed growth and exogenous pH profile patterns were typical of *C. acetobutylicum* [32].

### Metabolite Profiling of *C. acetobutylicum* ATCC824 and the Engineered Mutant Strain DG1

**Fig. 2:**
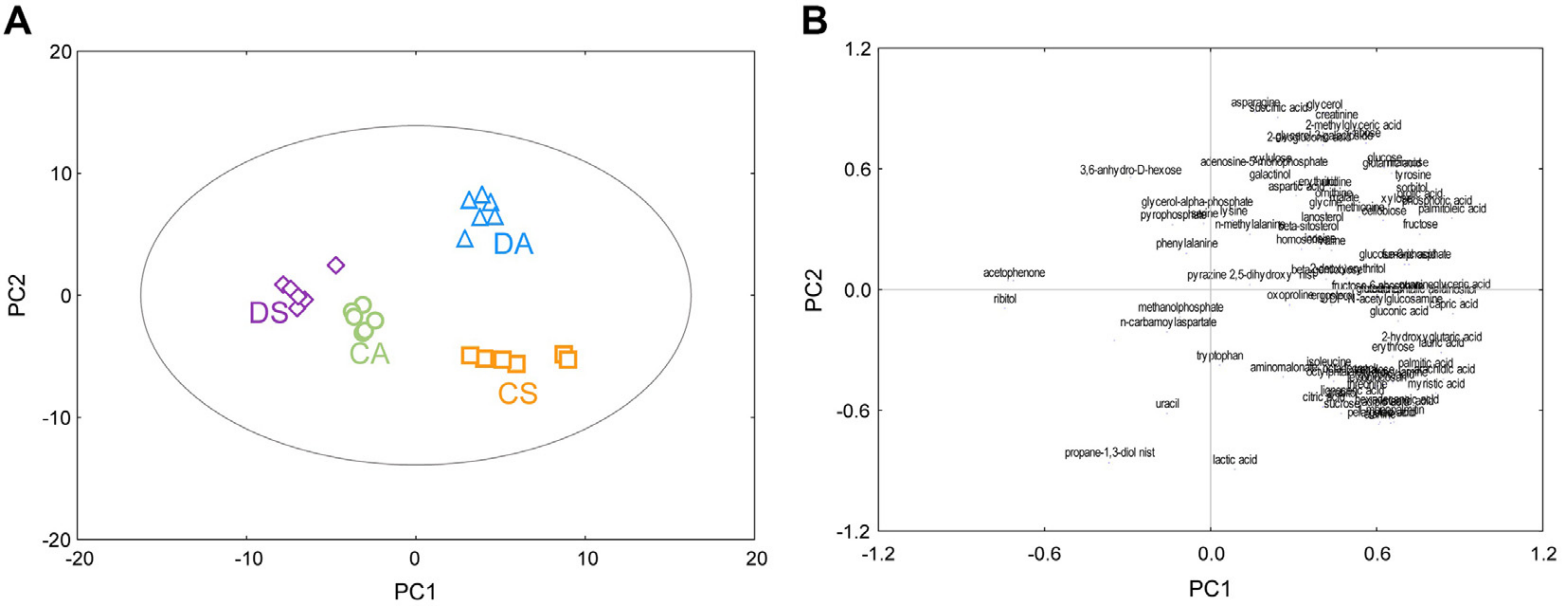
Principal component analysis (A) score plot and (B) loading plot of metabolites extracted from *Clostridium acetobutylicum* ATCC824 (wild type) and *C. acetobutylicum* DG1 (engineered strain) during the acidogenic and solventogenic phases. CA: *C. acetobutylicum* ATCC824 at acidogenic phase; CS: *C. acetobutylicum* ATCC824 at solventogenic phase; DA: DG1 mutant at acidogenic phase; DS: DG1 mutant at solventogenic phase.

Samples were collected during the acidogenic and the solventogenic phases from *C. acetobutylicum* ATCC824 and its engineered mutant strain DG1. The samples were cultured in six different batches. In total, 24 samples from four different classes in six replicates, including *C. acetobutylicum* ATCC824 and DG1, were analyzed during the acidogenic and solventogenic phases by performing GC/TOF MS. Over 1,000 unique mz^−1^ values with respective retention times were detected. Deconvolution and alignment using ChromaTOF and Binbase, respectively, yielded 92 metabolites (Table 1), which were classified into various chemical classes, such as amines, amino acids, fatty acids, organic acids, phosphates, sugars, and intermediates of all major pathways, including glycolysis, the tricarboxylic acid (TCA) cycle, amino acid metabolism, and fatty acid metabolism. To provide a comparative interpretation of the changes in the metabolic profiles of *C. acetobutylicum* ATCC824 and DG1 during the acidogenic and solventogenic phases, principal component analysis (PCA) was performed as an unsupervised evaluation using Statistica. The PCA results showed significantly different metabolite profiles of *C. acetobutylicum* and DG1 in the acidogenic and solventogenic phases (Fig. 2A). PCA model parameters for an explained variation (R^2^X) value of 0.51 and a predictive capability (Q^2^) value of 0.59 were significantly high, which indicated the quality and suitability of the model. PC2 separated the metabolite profiles of *C. acetobutylicum* ATCC824 and its engineered mutant strain DG1, whereas PC1 affected the separation of the acidogenic and solventogenic phases in both strains. In particular, the changes in metabolite profiles associated with the acidogenic–to-solventogenic transition in *C. acetobutylicum* ATCC824 shifted from negative PC1 to positive PC1; however, in the engineered mutant strain DG1, the opposite shift was observed. As shown in Fig. 2B, the loading plot indicated the contribution of each metabolite to PC1 and PC2. Among the 92 metabolites, 81 metabolites, including glyceric acid, myoinositol, capric acid, palmitoleic acid, arachidic acid, and lauric acid, contributed positively to PC1, whereas the remaining 11 metabolites, including ribitol, acetophenone, uracil, glycerol-phosphate, serine, and 3,6-anhydro-D-hexose, contributed negatively to PC1. In the loading plot of PC2, 46 metabolites, including lactic acid, alanine, oleic acid, and pelargonic acid, positively contributed to PC2, whereas 55 metabolites, including asparagine, glycerol, succinic acid, creatinine, 2-methylglyceric acid, and ribose, negatively contributed to PC2.

**Fig. 3:**
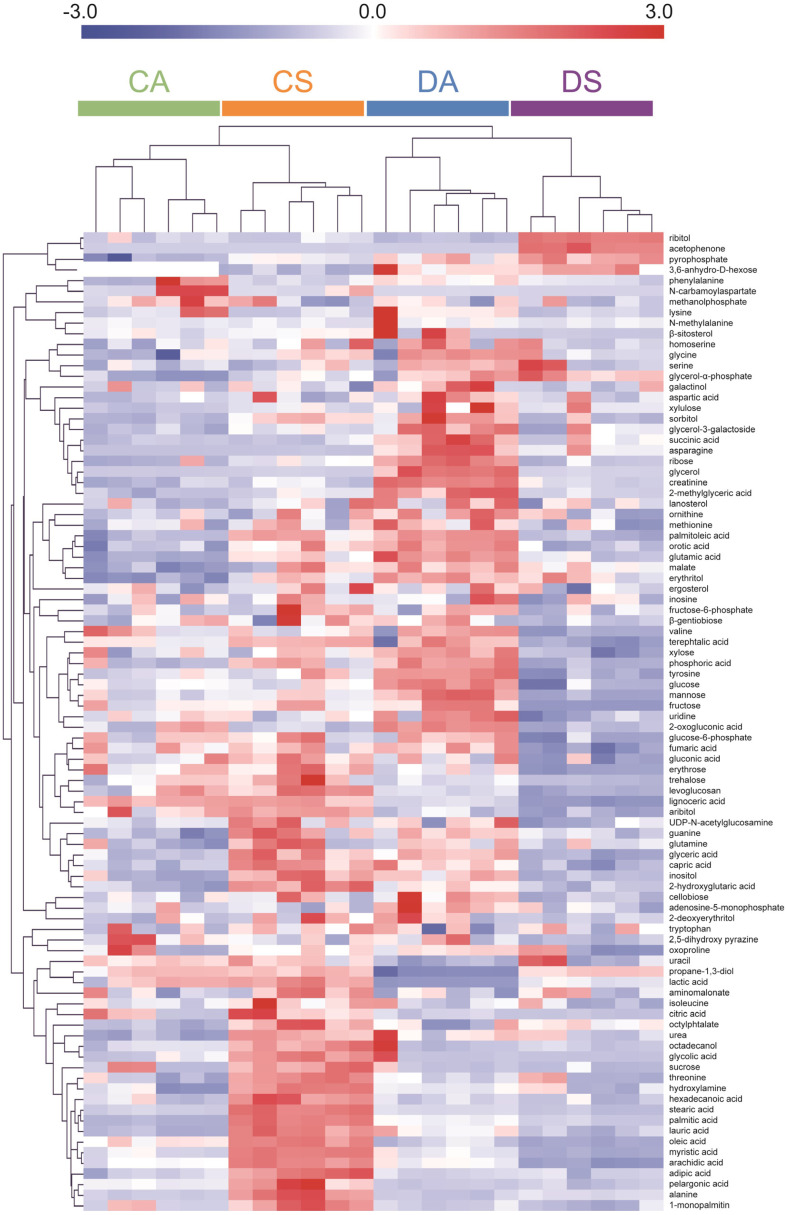
Hierarchical clustering analysis and heat map of metabolites extracted from *Clostridium acetobutylicum* ATCC824 (wild type) and *C. acetobutylicum* DG1 (engineered strain) during the acidogenic and solventogenic phases. CA: *C. acetobutylicum* ATCC824 at acidogenic phase; CS: *C. acetobutylicum* ATCC824 at solventogenic phase; DA: DG1 mutant at acidogenic phase; DS: DG1 mutant at solventogenic phase.

For the four classes, including *C. acetobutylicum* ATCC824 and DG1 in the acidogenic and solventogenic phases, the identified metabolites were clustered and visualized by performing HCA using the Euclidean distance coefficient and average linkage method [33]. A heatmap of the classes and identified metabolites is presented in Figure 3. After normalizing the raw data to the dry weight of the cell and transforming it by unit variance scaling, the data were analyzed using the TM4 software. The higher intensity of the metabolites was depicted in red in the heatmap, whereas the lower intensity was depicted in blue in the heatmap. The metabolite profiles of the six replicates in each group were similar. Conversely, the metabolite profiles differed significantly among the classes; however, the metabolite profiles of the two strains during the acidogenic and solventogenic phases were highly similar. A previous study reported that metabolite profiles changed according to the acidogenic-solventogenic transition in a wild type strain [17]. The present results are consistent with those of the previous study. The levels of specific metabolites were differenced among four classes, indicating the variations in the metabolic profiles of each class. For instance, the levels of phenylalanine and *N*-carbamoylaspartic acid increased significantly in the wild type strain during the acidogenic phase. The levels of metabolites such as adipic acid, stearic acid, palmitic acid, lauric acid, and oleic acid increased in the wild type strain during the solventogenic phase. The levels of some metabolites, including glucose, glutamic acid, aspartic acid, succinic acid, asparagine, ribose, and malic acid, were higher in DG1 than in the wild type strain during the acidogenic phase. Moreover, the levels of threonine, ribitol, and pyrophosphate were higher in DG1 than in the wild type strain during the solventogenic phase. These results implied that the changes in the metabolite profiles were affected by phase and strain transitions.

### Changes in the Intracellular Metabolite Levels in *C. acetobutylicum* ATCC824 and DG1 at Different Phases

Following the PCA and HCA analyses, the metabolite profiles of *C. acetobutylicum* ATCC824 and DG1 were altered during the acidogenic-to-solventogenic transition. A Student’s t-test at the 95% significance level was used to compare alterations in the two during this transition (Table 2). In *C. acetobutylicum* ATCC824, the levels of 41 metabolites, including palmitic acid, stearic acid, myristic acid, lauric acid, arachidic acid, and urea, increased during the transition. In the *DG1* mutant, the levels of six metabolites, including ribitol, lactic acid, alanine, and pyrophosphate, increased, whereas those of 52 metabolites, including lignoceric acid, arachidic acid, palmitoleic acid, myristic acid, and palmitic acid, decreased significantly during the transition. In particular, the levels of 22 metabolites, namely terephthalic acid, stearic acid, sorbitol, phosphoric acid, palmitoleic acid, palmitic acid, orotic acid, oleic acid, myristic acid, malic acid, lauric acid, myoinositol, guanine, glycerol-3-galactoside, glycerol, glyceric acid, glutamic acid, capric acid, asparagine, arachidic acid, 2-methylglyceric acid, and 2-hydroxyglutaric acid, increased in *C. acetobutylicum* ATCC824, whereas the opposite trend was observed for DG1.

## Discussion

This is the first metabolomics study on *C. acetobutylicum* DG1 to investigate the metabolism and biological functions of the strain using GC/TOF MS. *C. acetobutylicum* DG1 was engineered by introducing the pSPD5 plasmid from *C. butylicum* for producing 1,3-propanediol [22, 24]. No solventogenic phase was observed in the case of fermentation using the mutant, unlike that in fermentation using the wild type *C. acetobutylicum* ATCC824 because the pH of the exogenous medium gradually decreased during growth. The development of *C. acetobutylicum* DG1 has been reported in several studies [22, 24]; however, the mechanism of DG1 remains unknown. The present metabolomics findings provided insights into *C. acetobutylicum* DG1 metabolism, especially in comparison with wild type *C. acetobutylicum* metabolism.

Amador-Noguez *et al*. [17] reported changes in the metabolism of *C. acetobutylicum* ATCC824 during the acidogenic-to-solventogenic transition using liquid chromatography-electrospray tandem MS and ^1^H-nuclear magnetic resonance. Fewer metabolites were identified using GC/TOF MS in the present study (92 metabolites) than those in the previous study (114 metabolites), and these intracellular metabolites of the wild type and mutant strains were classified into various chemical classes, such as amines, amino acids, fatty acids, organic acids, phosphates, and sugars. The identification of fatty acids, including stearic acid, adipic acid, myristic acid, and lauric acid, which were not identified in the previous study, provided insights into the metabolic traits of fatty acid metabolism.

**Fig. 4:**
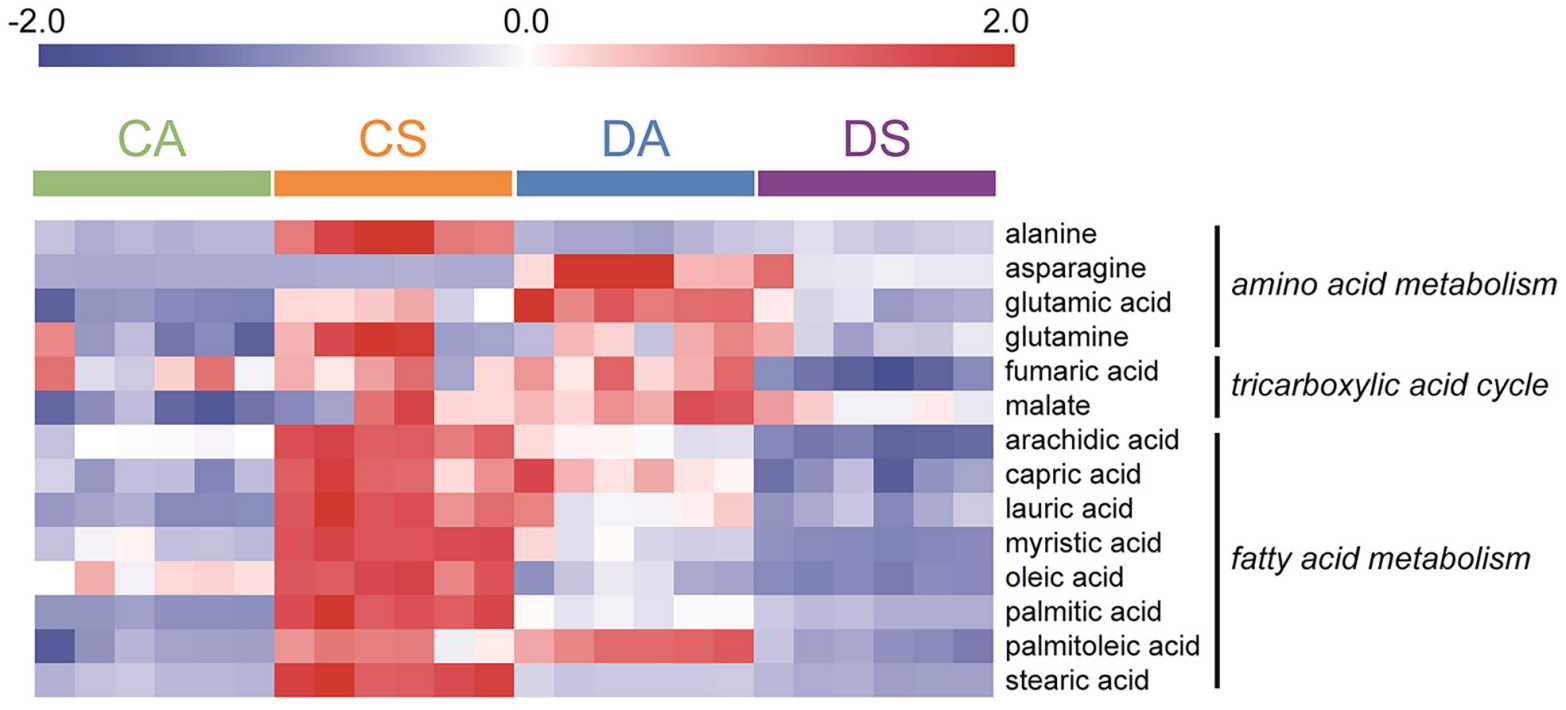
Intensities of metabolites related to amino acid metabolism, tricarboxylic acid cycle, and fatty acid metabolism. CA: *C. acetobutylicum* ATCC824 at acidogenic phase; CS: *C. acetobutylicum* ATCC824 at acidogenic phase ATCC824 at solventogenic phase; DA: DG1 mutant at acidogenic phase; DS: DG1 mutant at solventogenic phase.

Herein, the levels of amino acids, such as alanine, asparagine, glutamic acid, glutamine, and glycine, were higher in the solventogenic phase than in the acidogenic phase of the wild type strain. However, in the mutant, the abundance of amino acids, including asparagine, glutamic acid, lysine, phenylalanine, and tyrosine, decreased during the acidogenic-to-solventogenic transition (Fig. 4). The amino acid metabolism of the wild type strain was the opposite of that of the mutant strain. Jones *et al*. performed a transcriptional analysis and demonstrated that genes related to the transport and metabolism of amino acids were more highly expressed in the solventogenic phase than in the acidogenic phase of the wild type strain [34]. Consistent with the transcriptome analysis, the present metabolomics analysis showed that the levels of the amino acids increased in the solventogenic phase of the wild type, whereas their levels in the mutant decreased. Various organisms, such as bacteria, yeast, and plants, produce phenylalanine and glutamate under stress conditions [35-37] because these metabolites are associated with the synthesis of phenolic compounds necessary for defense against stresses. The production of solvents during the solventogenic phase is a major stress for *C. acetobutylicum* [38, 39]. In the present study, the levels of these amino acids increased during the solventogenic phase to reduce stress in the wild type strain. These results showed that the metabolism of amino acids was different between the wild type and mutant strains and that the formation of solvents did not act as a major stress in the case of DG1.

Previous studies have established that *C. acetobutylicum* undergoes a bifurcated TCA cycle, which involves oxidative and reductive directions depending upon the intracellular redox balance. In oxidative and reductive reactions, oxaloacetate is converted into succinate by forming the intermediates citrate/α-ketoglutarate and malate/fumarate, respectively [40, 41]. In this study, malate and fumarate levels were higher in the wild type strain during the solventogenic phase than during the acidogenic phase. Conversely, lower levels were observed in the mutant. The oxidative TCA cycle was more activated in the solventogenic phase in the mutant strain than in the wild type of strain (Fig. 4). Additionally, the direction of the TCA cycle differed between the wild type and the mutant.

The clostridial form is generally responsible for solvent production and is distinguished morphologically as a swollen cell with a bright granulose phase within the cell [42]. Previous studies have reported that solvent production generated stress conditions for *Clostridium* because of changes in the exogenous pH or the formation of solventogenic n-butanol [43-45]. In response to n-butanol stress, *C. acetobutylicum* ATCC824 modified its cytoplasmic membrane composition by increasing metabolic flux through the glycerolipid biosynthetic pathway [45]. Additionally, under ethanol stress, various genes involved in fatty acid, lipid, and isoprenoid metabolism were upregulated in *Saccharomyces cerevisiae* [46, 47]. Consistent with the transcriptional analysis, the present metabolomics data showed that the levels of fatty acids in the wild type strain in the solventogenic phase were higher than those in the acidogenic phase. However, the opposite trend was observed for the engineered strain (Fig. 4). The increased fatty acid level altered the membrane composition, which eventually affected the change in exogenous pH and the production of the solvent in the solventogenic phase of the wild type strain. These results indicated that solvent production did not act as stress for the mutant, although it altered fatty acid metabolism.

In conclusion, the amino acid and fatty acid metabolism and the TCA cycle of *C. acetobutylicum* ATCC824 and its engineered strain DG1 were significantly different. In the DG1 strain, the levels of amino and fatty acids were lower during the solventogenic phase than those during the acidogenic phase. Additionally, the abundance of malic and fumaric acids was lower in the solventogenic phase than in the acidogenic phase, indicating the direction of the oxidative TCA cycle, *i.e.*, oxaloacetate was converted into succinate by forming the intermediate citrate/α-ketoglutarate. These data suggest that global metabolite profiling is useful for understanding and identifying the metabolism and metabolic traits of microorganisms with unknown action mechanisms.

## Figures and Tables

**Table 1:** Intracellular metabolites extracted from *C. acetobutylicum* ATCC824 and DG1 mutant.

Class	Metabolites	CA^a^	CS^b^	DA^c^	DS^d^
Amine (8)	Creatinine	549.27±192.37	773.82±224.31	1984.23±130.42	792.98±159.12
	Guanine	92.88±44.47	267.90±86.65	217.31±40.18	141.49±30.26
	Hydroxylamine	103282.2±40698.11	223563.25±17545.27	137600.80±7161.33	124363.23±36113.72
	Inosine	47.39±19.79	55.06±18.13	60.56±27.13	42.04±20.09
	2,5-Dihydroxy pyrazine	110.89±56.52	61.97±28.52	84.38±47.82	34.11±9.79
	UDP-Nacetylglucosamine	31.31±4.15	51.23±18.32	42.79±20.34	22.40±10.32
	Uracil	171.24±17.87	174.71±49.66	47.99±24.90	148.42±145.42
	Uridine	49.20±8.66	44.42±10.78	71.71±21.68	25.82±12.33
Amino acid (20)	Alanine	1141.15±122.58	4732.29±1064.76	1024.76±226.48	1483.72±125.90
	Asparagine	363.06±57.60	518.30±156.12	10653.75±5038.12	4370.83±2912.32
	Aspartic acid	54.18±12.62	73.84±45.55	105.12±40.78	67.84±29.57
	Glutamic acid	857.93±88.64	1359.29±127.60	1825.45±192.51	1121.49±144.89
	Glutamine	102.99±61.11	206.82±93.05	168.60±42.04	135.63±33.65
	Glycine	13423.15±4171.27	18061.24±1682.74	20362.66±5274.90	16623.04±3148.54
	Homoserine	32.95±9.26	44.05±17.33	48.29±19.36	37.75±12.53
	Isoleucine	451.56±113.48	839.24±458.07	463.20±242.88	338.21±269.97
	Lysine	1789.94±1646.32	599.99±351.65	2223.99±1714.65	508.65±279.66
	Methionine	33.77±14.73	43.57±15.32	58.34±19.11	27.00±16.83
	N-carbamoylaspartate	1419.74±1495.22	394.90±514.18	62.61±13.90	23.28±4.69
	N-methylalanine	434.86±127.73	388.30±203.07	1718.27±2685.98	410.02±166.41
	Ornithine	99.77±15.81	112.52±41.08	150.31±32.22	99.12±32.36
	Oxoproline	8327.89±4861.65	8472.80±802.29	8658.23±897.51	6088.78±4242.44
	Phenylalanine	873.70±891.70	417.76±97.90	702.03±93.14	451.79±85.79
	Serine	475.28±227.66	550.82±307.49	940.35±336.54	855.96±712.99
	Threonine	420.86±242.72	1262.19±132.84	562.72±119.05	552.60±408.33
	Tryptophan	42.30±24.73	54.40±12.05	36.82±25.19	47.62±16.38
	Tyrosine	1475.78±251.05	1754.37±487.57	2591.41±196.89	858.22±289.24
	Valine	986.60±428.11	803.54±204.03	1056.46±432.87	299.91±97.55
Fatty acid (17)	1-Monopalmitin	219.16±86.51	423.00±97.63	166.42±27.68	124.95±45.96
	Adipic acid	12197.00±1905.06	24548.23±3087.85	12932.26±768.29	12401.63±1791.31
	Arachidic acid	23228.40±1603.95	36067.69±1848.22	23756.09±1514.90	12925.57±1357.15
	Capric acid	908.07±73.60	1358.86±137.08	1202.39±147.83	814.92±99.92
	Ergosterol	28.81±10.30	39.95±15.35	36.01±11.38	26.09±12.25
	Hexadecanoic acid	7731.32±1780.47	14768.12±2171.49	7677.78±804.93	6962.84±1390.20
	Lanosterol	21.97±9.75	29.30±13.80	39.42±15.48	24.24±10.95
	Lauric acid	2877.88±199.10	5748.83±503.31	4192.69±544.01	3105.86±334.99
	Lignoceric acid	8906.31±614.73	8924.22±1047.24	4981.23±308.63	2140.78±250.02
	Myristic acid	34745.30±4168.37	64913.10±1394.76	37521.23±3884.42	23087.30±981.74
	Octadecanol	16020.70±2381.79	30113.36±4132.39	18304.68±13056.55	14739.87±1536.08
	Oleic acid	2491.29±300.13	4104.45±347.11	1535.41±422.13	812.89±90.19
	Palmitic acid	190918.00±9080.78	520663.08±29186.23	301257.68±12577.39	238205.79±13921.82
	Palmitoleic acid	177319.78±20896.35	266122.11±29592.15	292702.16±16887.79	180963.23±14799.25
	Pelargonic acid	9115.50±487.25	19121.96±7087.99	6038.56±309.89	5928.03±1396.35
	β-Sitosterol	994.62±425.91	1322.09±169.50	2678.33±3060.65	207.22±66.65
	Stearic acid	1508757.57±80096.08	3778144.83±252400.52	1648973.60±49102.00	1315927.57±107443.35
Organic acid (14)	2-Hydroxyglutaric acid	123.64±46.81	368.64±56.33	231.35±32.63	141.28±29.31
	2-Methylglyceric acid nist	103.14±32.47	171.49±34.56	419.76±97.35	145.46±22.75
	2-Oxogluconic acid nist	512.89±319.36	256.05±83.06	921.91±62.39	162.91±95.52
	Aminomalonate	55.77±27.15	85.47±27.76	45.70±19.71	63.27±24.82
	Citric acid	763.49±666.83	1391.07±733.93	320.13±112.48	207.11±39.25
	Fumaric acid	499.09±70.33	502.80±66.93	539.99±51.30	325.62±36.76
	Gluconic acid	187.03±53.50	232.20±38.76	191.20±60.61	107.13±54.01
	Glyceric acid	737.25±168.16	1668.22±395.67	1411.72±215.23	516.67±116.24
	Glycolic acid	1002.90±859.15	4932.74±749.42	1515.62±2827.49	292.13±32.20
	Lactic acid	262999.90±96346.96	352967.71±47875.18	7914.08±3914.23	167184.23±23169.36
	Malate	243.85±65.34	473.04±163.78	566.51±81.52	455.52±50.80
	Orotic acid	247.11±38.22	335.11±37.86	384.44±22.47	259.27±30.04
	Succinic acid	112.21±33.50	141.33±63.85	554.26±199.73	234.64±134.46
	Terephtalic acid	917.96±91.11	1245.51±52.09	1083.96±510.03	411.14±107.90
Phosphate (8)	Adenosine-5-monophosphate	34.24±11.26	28.36±10.15	57.73±22.86	29.47±12.19
	Fructose-6-phosphate	33.15±12.31	59.53±33.06	47.93±20.44	29.35±13.51
	Glucose-6-phosphate	3200.72±1025.49	3544.16±1358.48	3731.97±1019.90	1201.32±790.85
	Glycerol-3-galactoside	71.89±28.87	129.38±27.61	270.37±85.21	127.80±94.33
	Glycerol-α-phosphate	130.69±88.82	333.72±70.48	466.22±206.87	575.50±159.00
	Methanolphosphate	45.54±21.23	26.90±25.38	27.68±15.58	23.52±15.83
	Phosphoric acid	98627.75±32208.56	144146.88±33852.00	179553.21±21895.74	78400.52±10685.78
	Pyrophosphate	2432.31±986.59	3978.10±299.43	4581.12±708.99	5585.63±563.67
Sugar (21)	2-Deoxyerythritol	185.86±103.88	310.41±162.36	305.98±175.99	158.24±27.85
	3,6-Anhydro-D-hexose	52.62±26.20	46.47±17.27	130.82±60.18	141.36±30.26
	Arabitol	544.57±205.91	645.36±39.99	322.17±78.17	199.93±68.71
	Cellobiose	63.58±24.21	89.20±50.69	124.05±54.44	40.61±15.19
	Erythritol	170.33±44.47	377.49±68.51	486.78±50.51	397.24±99.59
	Erythrose	459.65±158.92	541.94±173.70	344.78±92.47	98.92±29.36
	Fructose	100495.45±37812.55	120032.26±40360.34	159921.41±55222.31	981.24±783.99
	Galactinol	51.86±23.37	45.32±20.94	81.63±34.35	45.24±18.24
	Glucose	3617982.10±458532.44	3706864.23±659257.37	6262992.54±530272.46	1865313.56±1282865.07
	Glycerol	9262.76±657.10	13266.33±2260.37	146350.73±37576.54	16494.41±2538.34
	Inositol	630.97±209.00	1282.59±216.79	1076.92±140.19	626.92±63.44
	Levoglucosan	646.94±273.22	903.26±193.53	469.98±31.99	273.33±52.35
	Mannose	3858.98±1546.18	5066.00±1293.68	10057.51±2667.74	721.69±756.12
	Ribitol	615.54±303.09	548.81±185.88	322.17±78.17	2029.43±69.09
	Ribose	538.54±710.49	1026.79±219.37	2451.07±419.82	548.27±310.34
	Sorbitol	687.45±85.62	1399.19±180.45	1736.27±541.85	805.43±307.66
	Sucrose	3176.42±3630.94	7118.67±1816.45	1602.31±1570.93	929.19±1033.18
	Trehalose	886.35±358.42	1470.71±827.56	637.26±135.54	356.84±41.54
	Xylose	214.32±93.49	224.05±62.06	326.22±37.07	103.24±51.66
	Xylulose	315.57±77.79	640.76±199.45	1530.04±1177.56	883.30±593.12
	β-Gentibiose	48.37±14.75	56.72±27.05	54.30±14.15	37.70±6.11
Others (4)	Acetophenone	7498.06±3177.89	9061.45±3835.07	2841.39±1886.72	628573.59±86891.05
	Octylphtalate	3684.64±660.71	9447.45±2451.82	4837.15±2804.95	6435.86±606.37
	Propane-1,3-diol	52421.36±6036.49	54119.94±4074.16	8984.38±4419.40	51082.52±3149.83
	Urea	1266.30±160.90	2359.09±176.85	2135.31±646.22	1733.55±329.04

^a^CA: The intensity of metabolite extracted from *C. acetobutylicum* ATCC824 at acidogenic phase; ^b^CS: The intensity of metabolite extracted from *C. acetobutylicum* ATCC824 at solventogenic phase; ^C^DA: The intensity of metabolite extracted from DG1 mutant at acidogenic phase; ^d^DS: The intensity of metabolite extracted from DG1 mutant at solventogenic phase

**Table 2:** The mean intensities and P-values of metabolites that were significantly changed during the acidogenic-to-solventogenic transition in *C. acetobutylicum* ATCC824 and DG1 mutant.

Metabolite	Acidogenic	Solventogenic	P-value
Increased metabolites during acidogenic-to-solventogenic transition in *C. acetobutylicum* ATCC824			
Palmitic acid	190918	520663.1	<0.001
Stearic acid	1508758	3778145	<0.001
Myristic acid	34745.3	64913.1	<0.001
Lauric acid	2877.882	5748.831	<0.001
Arachidic acid	23228.4	36067.69	<0.001
Urea	1266.298	2359.092	<0.001
Sorbitol	687.4487	1399.195	<0.001
Oleic acid	2491.286	4104.454	<0.001
Glycolic acid	1002.895	4932.745	<0.001
Adipic acid	12197	24548.23	<0.001
Alanine	1141.149	4732.294	<0.001
2-Hydroxyglutaric acid	123.6387	368.6417	<0.001
Glutamic acid	857.9267	1359.293	<0.001
Terephtalic acid	917.9621	1245.511	<0.001
Threonine	420.8636	1262.187	<0.001
Octadecanol	16020.7	30113.36	<0.001
Capric acid	908.0675	1358.862	<0.001
Hydroxylamine	103282.2	223563.3	<0.001
Erythritol	170.3339	377.4909	<0.001
Hexadecanoic acid	7731.318	14768.12	<0.001
Palmitoleic acid	177319.8	266122.1	<0.001
Octylphtalate	3684.639	9447.447	<0.001
Glyceric acid	737.2467	1668.223	<0.001
Inositol	630.9663	1282.595	<0.001
Guanine	92.8791	267.9005	0.001
Glycerol-α-phosphate	130.6909	333.724	0.001
Glycerol	9262.763	13266.33	0.002
Orotic acid	247.1073	335.1097	0.002
1-Monopalmitin	219.1609	423.0002	0.003
Xylulose	315.5692	640.7635	0.004
Pyrophosphate	2432.308	3978.103	0.004
2-Methylglyceric acid	103.1389	171.4883	0.005
Glycerol-3-galactoside	71.89174	129.3816	0.005
Pelargonic acid	9115.498	19121.96	0.006
Malate	243.8488	473.0423	0.010
UDP-N-acetylglucosamine	31.30627	51.22877	0.027
Glycine	13423.15	18061.24	0.030
Phosphoric acid	98627.75	144146.9	0.038
Sucrose	3176.416	7118.674	0.039
Asparagine	363.06	518.3008	0.045
Glutamine	102.9886	206.8217	0.045
Increased metabolites during acidogenic-to-solventogenic transition in *DG1 mutant*			
Ribitol	322	2029	<0.001
Propane-1,3-diol	8984	51083	<0.001
Acetophenone	2841	628574	<0.001
Lactic acid	7914	167184	<0.001
Alanine	1025	1484	0.001
Pyrophosphate	4581	5586	0.022
Decreased metabolites during acidogenic-to-solventogenic transition in *DG1 mutant*			
Lignoceric acid	4981	2141	<0.001
2-Oxogluconic acid	922	163	<0.001
Creatinine	1984	793	<0.001
Arachidic acid	23756	12926	<0.001
Palmitoleic acid	292702	180963	<0.001
Tyrosine	2591	858	<0.001
Phosphoric acid	179553	78401	<0.001
Glyceric acid	1412	517	<0.001
Ribose	2451	548	<0.001
Myristic acid	37521	23087	<0.001
Xylose	326	103	<0.001
Glycerol	146351	16494	<0.001
Fumaric acid	540	326	<0.001
Mannose	10058	722	<0.001
Palmitic acid	301258	238206	<0.001
Orotic acid	384	259	<0.001
Levoglucosan	470	273	<0.001
Glucose	6262993	1865314	<0.001
Inositol	1077	627	<0.001
Glutamic acid	1825	1121	<0.001
Fructose	159921	981	<0.001
Stearic acid	1648974	1315928	<0.001
2-Methylglyceric acid	420	145	<0.001
N-Carbamoylaspartate	63	23	<0.001
Erythrose	345	99	<0.001
Capric acid	1202	815	<0.001
2-Hydroxyglutaric acid	231	141	0.001
Trehalose	637	357	0.001
Phenylalanine	702	452	0.001
Glucose-6-phosphate	3732	1201	0.001
Uridine	72	26	0.001
Valine	1056	300	0.002
Lauric acid	4193	3106	0.002
Oleic acid	1535	813	0.002
Guanine	217	141	0.004
Sorbitol	1736	805	0.004
Cellobiose	124	41	0.005
Succinic acid	554	235	0.009
Terephtalic acid	1084	411	0.010
Methionine	58	27	0.013
Arabitol	322	200	0.016
Malate	567	456	0.018
Glycerol-3-galactoside	270	128	0.021
Ornithine	150	99	0.021
Adenosine-5-monophosphate	58	29	0.023
Asparagine	10654	4371	0.025
β-Gentibiose	54	38	0.025
Gluconic acid	191	107	0.030
Pyrazine 2,5-dihydroxy	84	34	0.030
Lysine	2224	509	0.036
Citric acid	320	207	0.042
Galactinol	82	45	0.045
